# Process Intensification for Enhanced Fluoride Removal and Recovery as Calcium Fluoride Using a Fluidized Bed Reactor

**DOI:** 10.3390/ijms25094646

**Published:** 2024-04-24

**Authors:** Arindam Sinharoy, Ga-Young Lee, Chong-Min Chung

**Affiliations:** Department of Environmental Science & Biotechnology, Jeonju University, Jeonju 55069, Republic of Korea; arindam.sinharoy004@gmail.com (A.S.); dlrkdud1007@jj.ac.kr (G.-Y.L.)

**Keywords:** calcium fluoride, fluoride removal, crystallization, two-stage FBR, CFD, fluidized bed reactor

## Abstract

This study explored the feasibility of fluoride removal from simulated semiconductor industry wastewater and its recovery as calcium fluoride using fluidized bed crystallization. The continuous reactor showed the best performance (>90% fluoride removal and >95% crystallization efficiency) at a calcium-to-fluoride ratio of 0.6 within the first 40 days of continuous operation. The resulting particle size increased by more than double during this time, along with a 36% increase in the seed bed height, indicating the deposition of CaF_2_ onto the silica seed. The SEM-EDX analysis showed the size and shape of the crystals formed, along with the presence of a high amount of Ca-F ions. The purity of the CaF_2_ crystals was determined to be 91.1% though ICP-OES analysis. Following the continuous experiment, different process improvement strategies were explored. The addition of an excess amount of calcium resulted in the removal of an additional 6% of the fluoride; however, compared to this single-stage process, a two-stage approach was found to be a better strategy to achieve a low effluent concentration of fluoride. The fluoride removal reached 94% with this two-stage approach under the optimum conditions of 4 + 1 h HRT combinations and a [Ca^2+^]/[F^−^] ratio of 0.55 and 0.7 for the two reactors, respectively. CFD simulation showed the impact of the inlet diameter, bottom-angle shape, and width-to-height ratio of the reactor on the mixing inside the reactor and the possibility of further improvement in the reactor performance by optimizing the FBR configuration.

## 1. Introduction

The sources of fluoride pollution can be both natural and anthropogenic, contributing to environmental concerns and potential health risks [1]. Natural sources include fluoride-rich minerals and rocks from which fluoride leaches into water sources due to natural causes [2]. However, compared to natural sources, human activities significantly contribute to fluoride contamination. Industrial processes, such as mining and mineral processing, as well as aluminum and phosphate production, release fluoride into the air and water [3]. Agricultural activities can also potentially release fluoride into the environment, especially when using phosphate-based fertilizers and pesticides [4]. Furthermore, burning fossil fuels—coal in particular—releases pollutants into the atmosphere that include fluoride, which can subsequently contaminate soil and water bodies [5]. In order to effectively manage and reduce fluoride pollution, it is necessary to have a thorough understanding of these diverse sources as well as how they interact with ecosystems and human health [6].

In this study, semiconductor industry wastewater is being focused on. The semiconductor industry is one of major sources of fluoride pollution, primarily through the use of certain chemicals and processes in semiconductor manufacturing [7]. Hydrofluoric acid (HF), one of the most commonly used chemical in semiconductor fabrication processes, and its improper handling and disposal can lead to environmental pollution. In addition, the industry also utilizes fluorinated gases, such as sulfur hexafluoride (SF6), which have strong greenhouse effects and contribute to environmental pollution [8]. Depending on the specific processes and chemicals utilized in semiconductor manufacture, the amount of fluoride present in effluent from the semiconductor industry might vary significantly [9]. Chemicals containing fluoride may be used during etching, cleaning, and polishing processes in the semiconductor manufacturing process [6]. 

The semiconductor industry’s own efforts to minimize its environmental impact include the development and implementation of cleaner technologies, recycling programs, and rigorous waste management practices [8,10]. Despite these efforts, proper disposal and handling of fluoride-containing chemicals remain important to reduce potential negative effects on the environment and surrounding communities. Exposure to excess amounts of fluoride can cause various adverse health effects in humans, such as skeletal fluorosis, thyroid problems, neurological problems, skin defects, cardiovascular problems, etc. [5,6]. Different technologies, such as adsorption, precipitation, crystallization, membrane processes (reverse osmosis, nanofiltration), electrocoagulation, electrodialysis, etc., have been predominantly used for fluoride removal [2]. Most of these technologies require large amounts of costly, corrosive chemicals and generate waste steam or sludge with a high fluoride content, necessitating secondary treatment and proper disposal [6]. Instead of simply disposing of these fluoride-rich waste streams, it would be more advantageous to explore possibilities for recovering and reusing them. Among these technologies, coagulation/precipitation of fluoride using calcium-based chemicals (Ca(OH)_2_ and CaCl_2_) is a common practice, including in the semiconductor industry [11]. However, the sludge produced from such a process contains a high moisture content and is difficult to reutilize without further processing [1]. 

Compared with precipitation, another novel process, crystallization using almost the same mechanism and chemicals, can provide a much better end product from fluoride-containing wastewater treatment, which can be readily utilized in other industries as raw materials. For this purpose, a fluidized bed reactor (FBR) containing silica seed was proposed to be used under a continuous mode of operation. Although there are previous studies on fluoride removal using fluidized bed crystallization, most of these studies are focused on batch experiments and involve optimization of process parameters [12,13]. There is a lack of studies on continuous operation, including long-term ones, which are very important for industrial application of this technology. Additionally, none of the previous studies have implemented a two-stage FBR system as a process improvement strategy for fluoride removal from wastewater, which is one of the novelties of this study. Another important parameter, i.e., the FBR configuration, is often overlooked when considering its potential to improve reactor performance. The main reason for this could be the requirement of a large amount of resources to build and test different reactor configurations. In this context, computational fluid dynamics (CFD) can be a great tool, using which different reactor configurations can be examined without the need for actual reactor setups. However, despite having detailed knowledge and widespread application in various industries, CFD has never been used to optimize the FBR configuration for effective mixing of reactants inside the FBR, targeting improvement in fluoride removal. 

This study explored fluoride removal from simulated semiconductor industry wastewater using the calcium fluoride crystallization process. Initially, a silica seed-containing FBR was used under the continuous mode of operation for fluoride removal. Characterization of the CaF_2_ crystals recovered from the continuous FBR was performed using different instrumental techniques. Different process improvement strategies, namely, addition of excess calcium and a two-stage FBR process, were also implemented in order to meet the effluent standard set for fluoride by various regulatory organizations. Finally, the effect of the FBR configuration on fluoride removal was studied using CFD to explore any possibility of further improvement in the process efficiency by modifying the conventional FBR.

## 2. Results and Discussion 

### 2.1. Continuous FBR Performance

#### 2.1.1. Fluoride Removal

**Figure 1:**
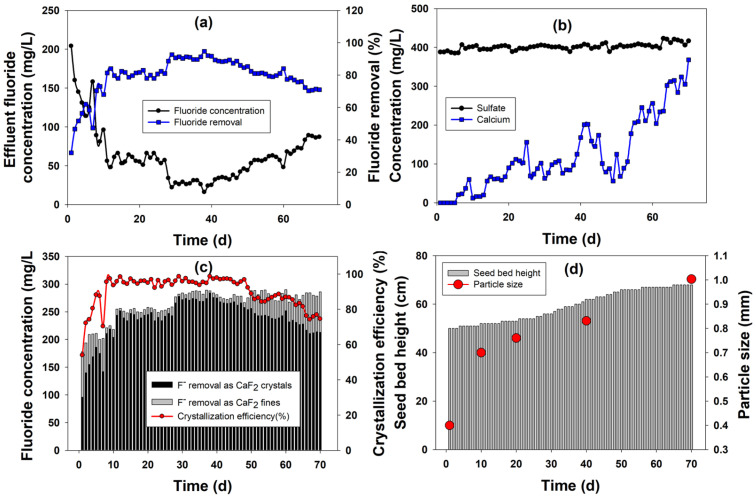
Fluoride removal and recovery as calcium fluoride during continuous FBR operation: (**a**) fluoride removal, (**b**) effluent calcium and sulfate profile, (**c**) calcium fluoride crystallization efficiency and (**d**) seed bed height and particle size profile during continuous operation.

The fluoride removal profile in the FBR during its continuous operation is depicted in Figure 1a. The influent fluoride concentration to the FBR was 300 mg/L. The initial fluoride removal efficiency was low at 32% on the first day, from which it grew to reach nearly 70% during the first 10 days of reactor operation. The respective effluent fluoride concentration varied between 81 and 204 mg/L (Figure 1a). During this initial operation, the Ca^2+^-to-F^−^ ratio was 0.55, which was based on the optimum ratio obtained during an earlier experiment [14]. However, as the desired low effluent fluoride concentration could not be achieved at this ratio and the calcium utilization was very high, the inlet calcium concentration was increased to make the Ca^2+^-to-F^−^ ratio 0.6 from 11th day onwards. With this increase in the influent Ca^2+^ concentration, a positive impact on fluoride removal could be observed. A consistent fluoride removal efficiency of 80.7 ± 1.8% could be achieved during the next 17 days of continuous reactor operation (Figure 1a). Consequently, the fluoride concentration in the reactor effluent lowered to 60 ± 6 mg/L during this time period. This positive impact of the inlet Ca^2+^ ion increase on fluoride removal is consistent with previous studies where a high inlet calcium value correlated with better fluoride removal performance [15]. Following this period, the reactor performance showed further improvement, with fluoride removal reaching 90.2 ± 1.7% during the following 22 days of FBR operation. The resulting fluoride concentration was 30 ± 8 mg/L during this period (28–50th day). From the 51th day onwards, the effluent fluoride concentration gradually increased as its removal efficiency depleted. This reduction in fluoride removal can be distinguished in two stages: for the first 10 days, the removal efficiency was 80.9 ± 1.4%, along with an effluent F^−^ concentration of 57.2 ± 4.2 mg/L, and for the remaining 10 days, these values were 73.9 ± 2.9% and 78 ± 8.9 mg/L, respectively (Figure 1a). The continuous reactor experiment showed that the FBR can successfully remove fluoride as calcium fluoride crystals; however, over a prolonged period of time without replacing the seeds, the performance declines. 

The fluoride removal performance obtained in this study is much superior to those reported in previous studies. For example, Aldaco et al. [16] obtained a maximum of 80% fluoride removal under the best conditions, which is nearly 10% less than the maximum fluoride removal value obtained in this current study. Even when the process efficiency of CaF_2_ crystallization is compared with other similar processes, such as fluorapatite (Ca_5_(PO_4_)_3_F) crystallization, the fluoride removal results obtained in this study are better. For instance, Deng et al. [17,18] reported >90% fluoride removal using fluorapatite crystallization with only 4.8 and 9.1 mg/L of influent fluoride concentrations. In comparison, the current study was able to remove ~81% fluoride from simulated wastewater containing 460 mg/L of influent fluoride concentration. 

#### 2.1.2. Calcium and Sulfate Profile during FBR Operation 

The calcium and sulfate profile during the continuous FBR operation is shown in Figure 1b. The calcium profile showed an increasing trend during the continuous operation. Initially, the calcium utilization was very high and the effluent calcium concentration ranged in between 12 and 60 mg/L. Following the increase in the influent calcium concentration to meet the demand of the crystallization reaction, the effluent calcium concentration also increased beyond 100 mg/L. There was a lot of fluctuations in the effluent Ca concentration during the later period of FBR operation, with periodic rises to as high as 150 mg/L or even 200 mg/L. The mean effluent calcium value during the 11–50th day period was 96.3 mg/L, with a high standard deviation (44.3). During the later period, when fluoride removal performance declined, a corresponding increase in the effluent calcium concentration could also be observed. The calcium concentration in the effluent stream remained above 200 mg/L from the 55th day onwards and above 300 mg/L from the 64th onwards, respectively. This increase in the calcium concentration during the later period of the FBR operation could be due to the accumulation of excess calcium from the recycle stream as well as from the continuous CaCl_2_ input to the reactor. Also, the gradual increase in the Ca concentration in the effluent, particularly during the later part of the reactor operation, corresponds well with the decrease in fluoride removal performance, indicating some form of limitation in the CaF_2_ crystallization reaction. This could be attributed to either the solubility of the reactants or their proper mixing, two key factors influencing the reaction; however, this will require further experiments to confirm. Moreover, it needs to be mentioned here that effluent Ca values ≈300 mg/L are common in industrial processes treating fluoride-containing wastewater, including calcium-based fluoride precipitation methods. Hence, from the treatment point of view, this is not unusual, but the recovery and utilization of these calcium ions from effluent should be explored to further improve the process economics. 

The sulfate concentration was also measured to understand its impact on fluoride removal through calcium fluoride crystallization. Sulfate is present as a co-pollutant in semiconductor industry wastewater [10], and it can interfere with CaF_2_ synthesis by forming calcium sulfate. However, in this study, no such interference was observed. The sulfate concentration in the FBR effluent remained almost the same (401.8 ± 8.2 mg/L) throughout the reactor operation (Figure 1b). This indicates that sulfate was not removed during FBR operation and did not interfere in the CaF_2_ crystallization process in any way. This is important because, in many cases, the formation of undesired CaSO_4_ can decrease the fluoride removal efficiency by reacting with the Ca ions present inside the reactor, and if precipitated along with CaF_2_, can reduce its purity as well.

#### 2.1.3. Crystallization Efficiency 

The fluoride speciation and CaF_2_ crystallization efficiency are shown in Figure 1c. The crystallization efficiency was initially low (53.9–88.1%) for first 7 days, following which the value consistently stayed above 95% during the next 41 days of continuous FBR operation. From the 50th day onwards, along with the reduction in the fluoride removal efficiency, the crystallization efficiency started to gradually decline. During this phase, except for the last 5 days of FBR operation, the crystallization efficiency was >80%. The maximum crystallization efficiency value obtained in the reactor was 98.6% on the 38th day of continuous reactor operation. The crystallization efficiency obtained in this study is comparable to or even higher than the values reported in the literature, indicating the superior performance of the continuously operated FBR. For example, Aldaco et al. [12] found the crystallization efficiencies to be within 69–75%, which is lower than the value obtained in this study (>80%). The fluoride speciation during the reactor operation corresponds well with the crystallization efficiency results. The portion of fluoride removed as calcium fluoride fines during the initial days of FBR operation was high (25–82 mg/L) compared with the next 42 days of FBR operation, during which the amount of calcium fluoride fines remained <20 mg/L or even <10 mg/L. During the initial time period (1–7 d), the amount of fluoride removed as CaF_2_ crystals was low (96–186 mg/L), indicating that although calcium fluoride formation was initiated in the reactor, the particles could not be retained on the silica seed materials and escaped the reactor along with the effluent. During the best-performing operating period (8–50th days) of the FBR, the concentration of CaF_2_ fines were much lower (<18 mg/L) than the initial phase, and the majority (204–284 mg/L) of the fluoride removed in the reactor went toward CaF_2_ crystallization (Figure 1c). However, as the FBR performance declined during the last phase of FBR operation in terms of the fluoride removal, the amount of fluoride removed as crystals also reduced (≈210 mg/L). Consequently, the amount of CaF_2_ fines increased to >60 mg/L during this last phase of FBR operation.

This increase in CaF_2_ fines in the later period of continuous reactor operation is not uncommon and can be explained in two ways. The calcium concentration increases along with the operating time, as can be seen from the calcium profile. This increase in the calcium concentration causes it to thicken and form scaling on the inner wall of the reactor. Some of the fluoride ions are captured in this calcium scaling, which does not precipitate onto the silica seed but rather comes out of the reactor as calcium fluoride fines [19]. The other reason is the breakdown of CaF_2_ scales from the deposited crystals formed over the silica seeds due to shearing and tearing by upflow fluidization during continuous reactor operation over an extended time period [14]. Therefore, proper care must be taken regarding the operating time of the FBR so that optimum fluoride removal along with ideal CaF_2_ crystal recovery can be achieved. 

The seed bed height was also monitored during the 70 days of continuous reactor operation and is shown in Figure 1d. As can be seen from the figure, the seed bed height grew consistently throughout the reactor operation, indicating the formation and growth of CaF_2_ crystals inside the FBR. The growth rate of the seeds was at its maximum during the 15 to 50th days of reactor operation, which correlate well with the time period during which the other reactor performance indicators, namely fluoride removal and crystallization efficiency, were at their best as well. The increase in the particle size of the seeds also showed a similar pattern, with 75%, 90%, 107.7% and 150.7% growth over a 10-, 20-, 40- and 70-day reaction period, respectively (Figure 1d). This increase in the particle size depends on many different factors, including the size of the seeds used, reaction time, etc. Aldaco et al. [12] reported a 70.6% and 76.3% increase in particle sizes for 250 µm (90 h) and 225 µm (110 h) seed sizes, respectively. 

#### 2.1.4. Characterization of Calcium Fluoride Crystals

**Figure 2:**
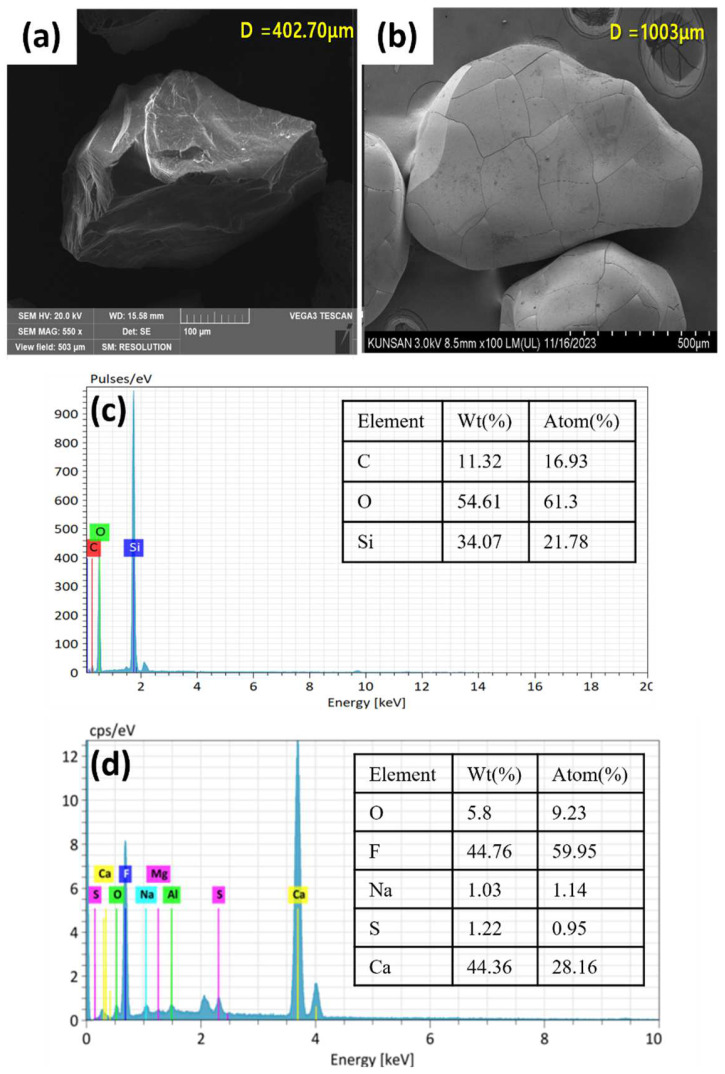
SEM images (**a**,**b**) and corresponding EDX spectra (**c**,**d**) of silica seed (**a**,**c**) and calcium fluoride crystals (**b**,**d**) obtained after 70 days of FBR operation.

Figure 2a,b depict the SEM images of the silica seed and CaF_2_ crystal after 70 days of continuous reactor operation. The crystal size increased by more than double from the 402.7 µm diameter of the initial silica seed to a 1003 µm diameter on the 70th day. The outer appearance of the crystal was smooth, and the shape seemed to be irregular but nearly spheroid. A similar shape of CaF_2_ crystal formed during fluidized bed crystallization has previously been reported [12,13]. The corresponding elemental composition of the crystal surface is provided by the EDX spectra of the initial silica seed and calcium fluoride crystals (Figure 2c,d). The initial seed contained Si, O and C, which when exposed to fluorine and calcium inside the FBR showed peaks for the presence of other elements, including F, Ca, Na, Al, Cl, and S, in addition to the original elements Si, C and O. The F and Ca composition in the 70-day-old CaF_2_ crystals was 44.8% and 44.4%, respectively. This indicates the extent of the deposition of CaF_2_ onto the silica seed. Furthermore, the presence of Si was not detected in the 70th-day sample, which could be due to the fact that the entire seed surface is covered with deposited CaF_2_ by such prolonged reactor operation. 

**Figure 3:**
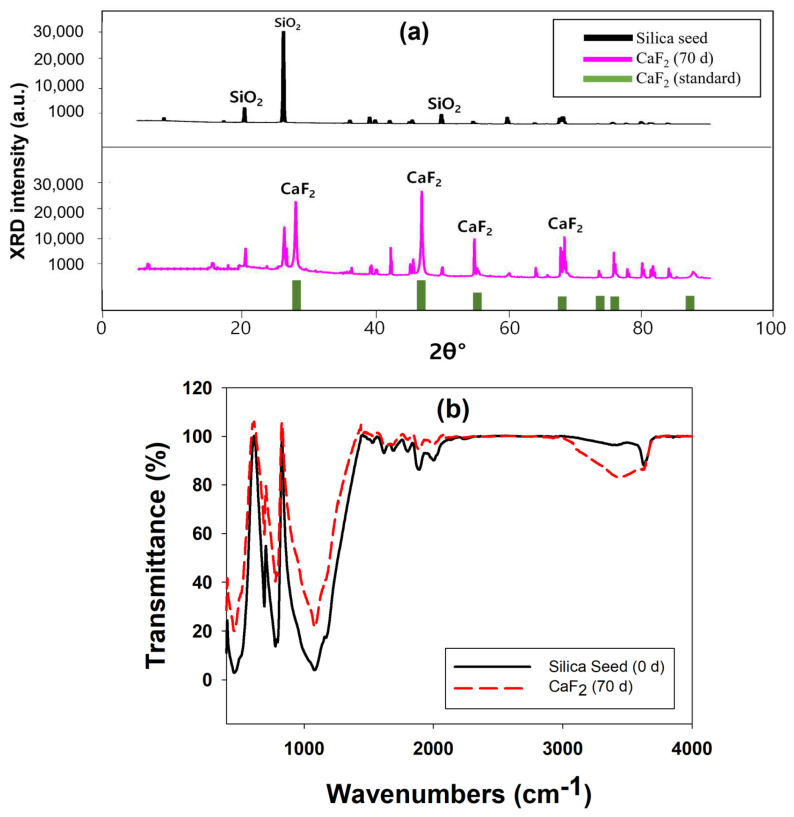
XRD (**a**) and FTIR (**b**) spectra of silica seeds and calcium fluoride crystals obtained after 70 days of FBR operation.

The XRD spectra of the calcium fluoride crystals formed inside the FBR are shown in Figure 3a. The original silica seed showed peaks at 2θ of 20.39°, 26.07° and 49.02°, which are typical of the SiO_2_ [20]. The characteristic peaks corresponding to the CaF_2_ were observed in the crystal samples obtained after 70 days of reactor operation. The peaks corresponding to CaF_2_ that are present in this sample were at 2θ of 28°, 46.6°, 55.7°, 68.7°, 75.8° and 87.4° (JCPDS no. 87-0971) [21,22]. Formation of CaF_2_ with the FBR was confirmed by this XRD analysis. The XRD characterization of the CaF_2_ crystals also revealed the absence of calcium carbonate (CaCO_3_), calcium sulfate (CaSO_4_) and fluorapatite (Ca_5_(PO_4_)_3_F) in the resulting crystals. These compounds are known to precipitate during fluoride removal through fluidized bed crystallization, and in some cases they are even the main product instead of CaF_2_ [18,20]. The possibility of any of these compounds’ formation in this study is completely eliminated through this XRD analysis, which also highlights the high purity of the CaF_2_ formed.

The FTIR spectra obtained for the silica seed material and calcium fluoride crystals obtained after 70 days of FBR operation are shown in Figure 3b. The broad peaks at 3450 cm^−1^ and 1620 cm^−1^ represent stretching and bending vibrations of the hydroxyl (-OH) group [23]. A range of small peaks at 1875 cm^−1^ and 2000 cm^−1^ could be due to the presence of C=O and C=C stretching vibrations, respectively [24]. The sharp peak at 1080 cm^−1^ indicates the presence of asymmetric stretching vibrations of the Si–O–Si bond [25]. The C=C bending vibration can also be seen via the peak at 830 cm^−1^ [26]. Other peaks at 750 cm^−1^ and 460 cm^−1^ can be attributed to the presence of sulfate and phosphate groups [23]. However, Si–O stretching vibration can also be indicated by the presence of a peak at 460 cm^−1^ [27]. Although it is difficult to confirm the presence of CaF_2_ with FTIR spectra alone, as it can only indicate the presence of functional groups, some of the previous studies [23,26] have suggested that the peak near 750 cm^−1^ is due to the presence of CaF_2_, which can be observed even in the current study. The change in certain peak intensities following exposure to fluoride indicates the role of this active group in the interaction with fluoride. For example, the peaks at 460, 830, 1080, 1875 and 2000 cm^−1^ showed reduced intensities for calcium fluoride crystals compared to silica seed, indicating the involvement of role-related functional groups such as Si–O, Si–O–Si, C=O and C=C in the interaction of fluoride ions with silica seeds. The increase in the peak intensities of the OH group at 3450 cm^−1^ for the CaF_2_ sample is due to the presence of water in the crystals due to prolonged exposure to experimental conditions inside the FBR [20]. 

Compositional analysis of the silica seed and CaF_2_ crystals obtained at the end of the reactor operation was carried out using ICP-OES, and the results are shown in Table 1. For this analysis, the samples were first acid-digested, and only the acid soluble parts were analyzed. It can be observed from the result that the calcium and fluoride composition increased significantly from 0.34% and 1.53% in the seed material to 33.69% and 57.37% after 70 days of FBR operation. This increase in the Ca and F composition of the samples directly corresponds to the reduction in the silica composition from (94.81% to 2.05%) and confirms the calcium fluoride crystal formation on the silica seed. Other elements such as sulfur, aluminum, iron, potassium, magnesium, sodium and phosphate are also present in the samples in minute quantities, which may have been contributed by either the silica seed material or by the simulated wastewater used in the study. The purity of the CaF_2_ crystals was also calculated in this study by converting the elemental percentages to molar fractions and subtracting the impurities from the CaF_2_ crystals (details shown in the Supplementary Material). By this method, the purity of the CaF_2_ crystals obtained after 70 days of FBR operation is 91.12%, which is relatively high compared to many other previous studies [14]. 

### 2.2. Process Improvement Strategies

#### 2.2.1. Effect of Increased Calcium Addition on Fluoride Removal

**Figure 4:**
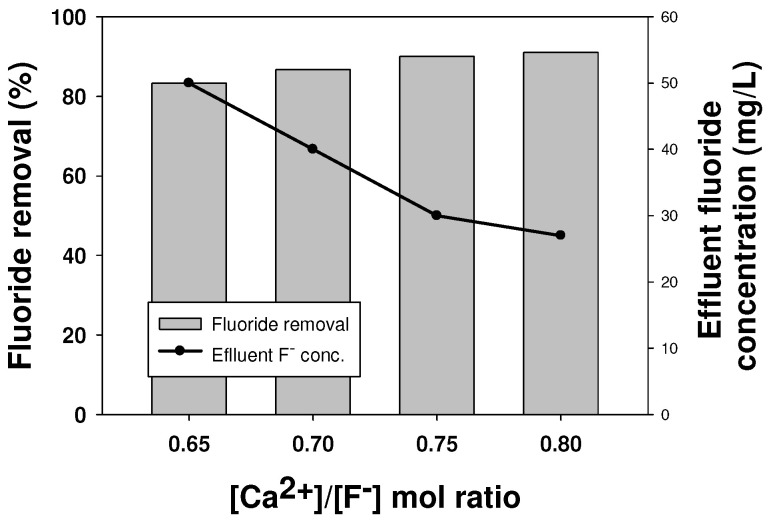
Fluoride removal performance at a high calcium-to-fluoride ratio.

In order to remove the additional fluoride present in the FBR effluent, an excess amount of calcium was injected into the reactor to reach a [Ca^2+^]/[F^−^] ratio of 0.65, 0.7, 0.75 and 0.8. The results showed that with an increase in the inlet calcium concentration, the effluent fluoride concentration gradually decreased (Figure 4). A minimum effluent fluoride concentration of 21 mg/L was obtained for a [Ca^2+^]/[F^−^] ratio of 0.8, which was 7% lower than that obtained originally for a [Ca^2+^]/[F^−^] ratio of 0.65 (Figure 4). The overall fluoride removal at this calcium concentration was 93%. It is to be noted here that it is impossible to remove more than 95% of fluoride due to the limitations associated with the solubility of calcium fluoride at a high concentration [28]. Hence, if calcium is not sufficiently dissolved, it affects its reaction with fluoride and its subsequent removal. In this case, the reaction solution is saturated and no additional calcium is dissolved, so the reaction rate with fluoride drops with any further increase in the influent calcium concentration. In addition, undissolved calcium can precipitate, which has been known to react with other inert substances in the solution to produce other undesired calcium compounds [6,19]. Therefore, to achieve a further lower effluent fluoride concentration by increasing the inlet calcium ions was not attempted in this study. 

#### 2.2.2. Performance of a Two-Stage FBR for Fluoride Removal

**Figure 5:**
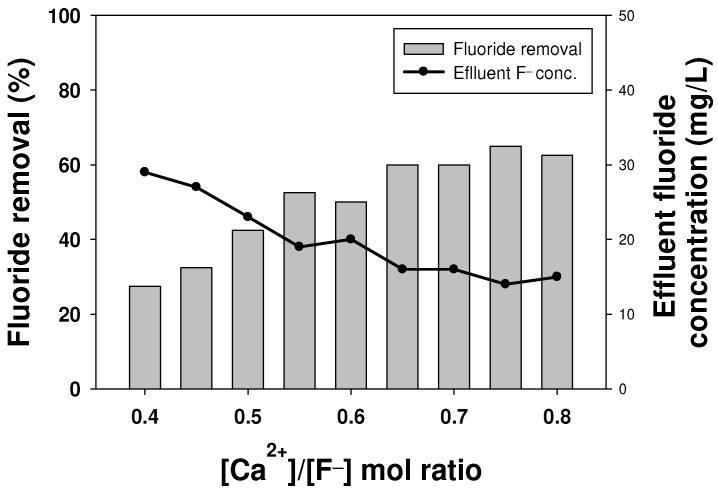
Effect of the calcium-to-fluoride ratio on fluoride removal in the second stage of the two-stage process.

In order to overcome the disadvantages of the single-stage process to achieve a low effluent fluoride concentration, a two-stage FBR process was designed. The two most important parameters, the [Ca^2+^]/[F^−^] ratio and hydraulic retention time (HRT), were optimized for this two-stage FBR. Figure 5 shows the effect of the [Ca^2+^]/[F^−^] molar ratio on fluoride removal in the second stage, which was fed with fluoride-containing (40 mg/L) wastewater coming out of the first reactor and had a fixed HRT of 1 h. The results showed that the fluoride removal increased with an increasing influent calcium concentration. The fluoride removal of 65%, along with an effluent fluoride concentration of 14 mg/L, was achieved at a [Ca^2+^]/[F^−^] ratio of 0.75 in the second stage. However, the effluent fluoride concentration as well as the fluoride removal efficiencies were almost similar for a [Ca^2+^]/[F^−^] ratio of 0.65–0.8. This observation is similar to the previous findings where an increase in the influent calcium concentration showed a positive impact on the fluoride removal due to the common ion effect, where an excessive amount of calcium ions compared with a low fluoride concentration improved the reaction rate for calcium fluoride formation [15].

**Figure 6:**
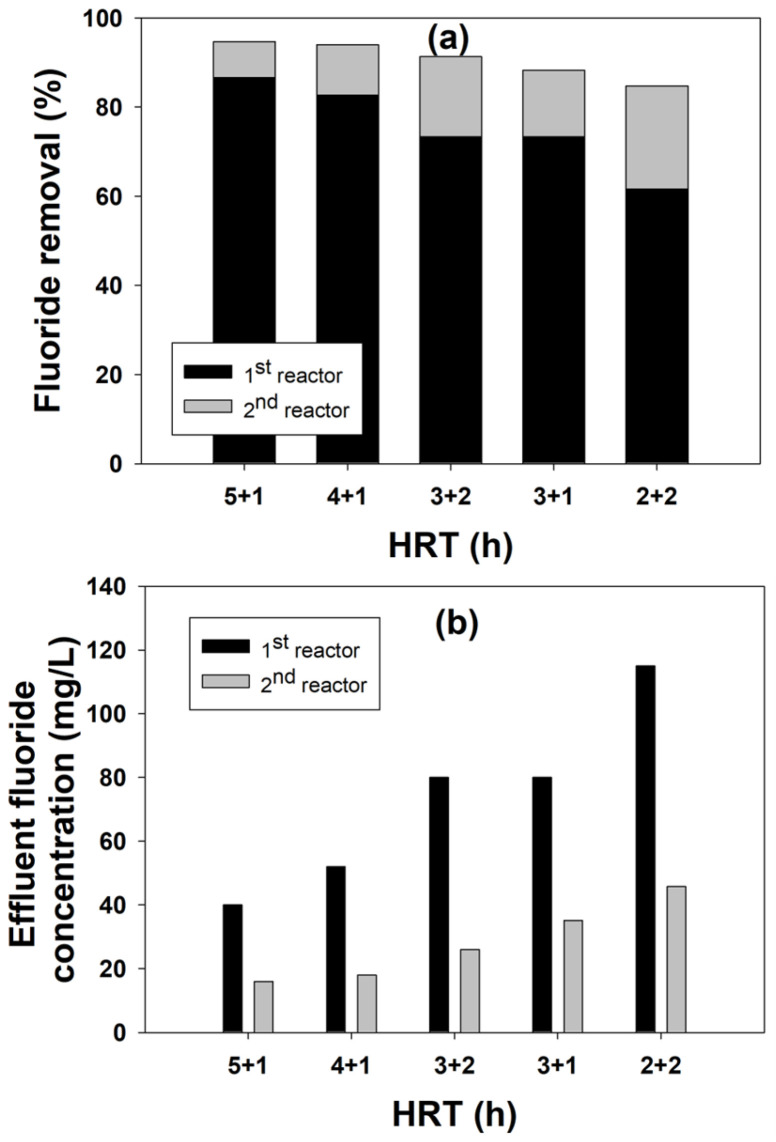
Effect of different HRT combinations on fluoride removal (**a**) and effluent fluoride concentration (**b**) in the two-stage FBR process.

The effect of different HRT combinations for the two-stage process on fluoride removal is shown in Figure 6. The goal of this experiment was to reduce the HRT without compromising the fluoride removal performance. A lower HRT is advantageous as more wastewater can be treated in a reactor of the same capacity, and it can be operated efficiently by reducing the installation and operating costs and optimizing energy consumption [29,30]. As can be seen from the figure, the 5 h + 1 h and 4 h + 1 h HRT for the first reactor and second reactor showed the best results, with 94.6% and 94% fluoride removal efficiency, respectively. However, considering the reduction in the HRT by 1 h of time in the 4 h + 1 h combination, this HRT should be considered optimum as it not only maintains the same level of fluoride removal performance but also reduces the overall reaction time. From the results, it is clear that the HRT has a profound effect on fluoride removal. The reduction in the HRT in the first reactor reduced its fluoride removal capacity, which could be overcome in the second reactor even with just a 1 h HRT. Such good performance in the second reactor is due to the presence of an excess amount of calcium ions and the relatively low influent fluoride concentration compared with the first reactor [11]. For the experimental conditions with a low HRT in the first stage (2 h and 3 h), the overall fluoride removal was not as good as the other two, despite having a high HRT of 2 h in the second stage. This could be attributed to the fact that this decrease in the fluoride removal efficiency was due to an insufficient reaction time between the reactants and the seeds in the reactor [14]. 

With the optimization of both these parameters, it can be conclusively stated that a two-stage process is better than a single-stage FBR process for fluoride removal. The addition of a second stage with fresh seeds provides for better fluoride removal opportunities as these additional seeds in the second stage increase the surface area available for calcium fluoride precipitation, thereby increasing the overall removal efficiency of the system [31]. Furthermore, by dividing the reaction for fluoride removal into two stages, the sensitivity to the reaction conditions can be reduced [32]. With this reduction in reaction sensitivity, the chemical reaction can be made relatively stable, even in an unstable environment, increasing the stability and efficiency of the overall operation.

### 2.3. CFD Simulation of an FBR Treating Fluoride Containing Wastewater

**Figure 7:**
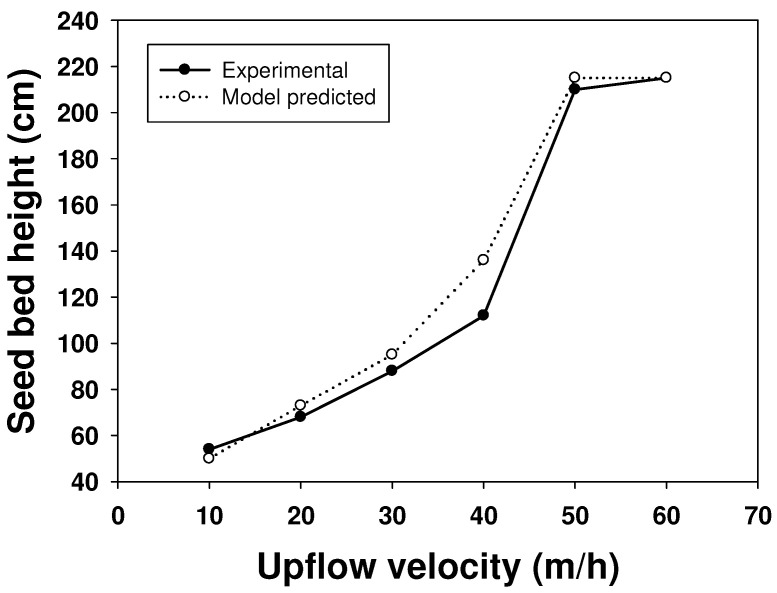
Comparative study of the experimental and CFD model-predicted seed bed height at different upflow velocities.

In order to verify the accuracy of the CFD model, attempts were made to check the similarity between the experimental and model-predicted increase in the seed bed height during fluidization with upflow velocities. The results of this experiment are shown in Figure 7. The results showed that there was a very good similarity between the experimental and model-predicted values for all the different upflow velocities except for 40 m/h, when a maximum 13% variation between the two results was observed. This indicates a consistent trend in terms of the CFD model in predicting the mixing pattern inside the reactor. The somewhat deviating results for certain values could be attributed to the difference between the actual experimental condition and the assumptions made regarding them during the CFD model development. For example, the shape and size of the particles were assumed to be spherical and 0.5 mm, respectively, for experimental simplicity. However, in actuality, the particles were of 0.3–0.7 mm in size and of an irregular shape with a similarity to a spheroid. 

In this study, CFD simulation was conducted to design a fluidized bed reactor for the purpose of maintaining the fluidized bed and proper mixing of crystals. By comparing the conditions at the bottom of the reactor with those of the existing reactor, an attempt was made to reduce the formation of dead zones by optimizing the angle. A dead zone refers to a location where no reaction occurs and instead remains stagnant, and this phenomenon significantly impacts a fluidized bed reactor. This absence of reactions within the dead zone leads to decreased seed activity, resulting in reduced efficiency, and lowers the reaction rate, thereby diminishing the overall system efficiency [33]. Additionally, the presence of dead zones within the reactor leads to non-uniform reactions, making it challenging to produce the desired crystal product, and it may compromise the process stability due to inefficient space utilization [34]. Therefore, minimizing the dead zones is crucial for operating the fluidized bed reactor under optimal conditions. 

**Figure 8:**
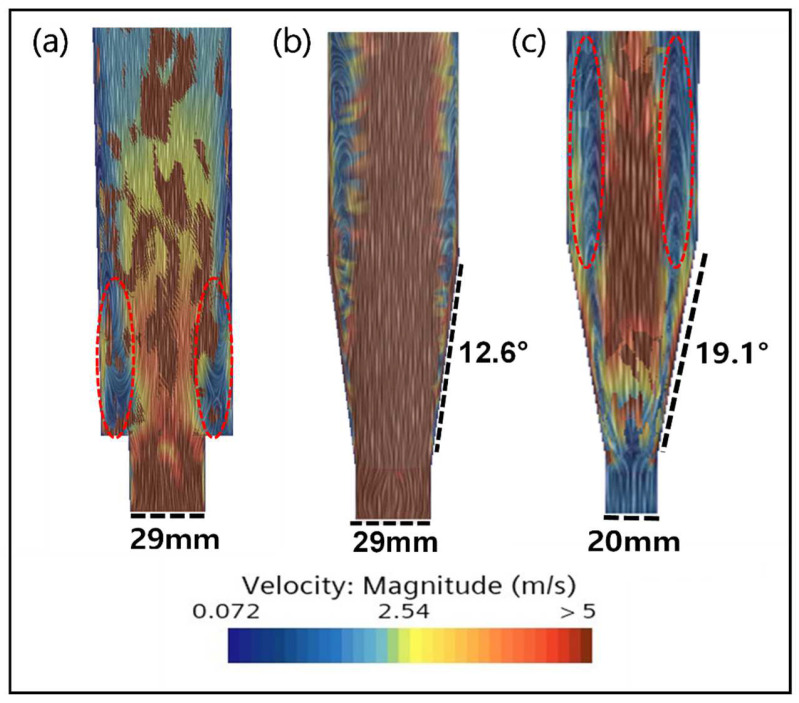
Simulation results of (**a**) the existing reactor with an inlet diameter of 29 mm, (**b**) the FBR with a lower angle of 12.6° and inlet diameter of 29 mm, and (**c**) the FBR with a lower angle of 19.1° and inlet diameter of 20 mm. Circles with red dotted lines show dead zones formed inside FBR.

The results of the CFD simulation for different FBR configurations are shown in Figure 8. From the figure, it is clear that there was formations of dead zones in the FBR with the original configuration, which reduced with the change in shape of the reactor to a conical bottom. In a cone-shaped structure, the diameter increases as it moves from the center of the lower part upwards. This contributes to maintaining the consistency of the reaction by minimizing the change in the reaction conditions depending on the distance from the center to the outermost point, thus reducing the non-uniformity of the entire reaction area and reducing the possibility of forming a dead zone [33,35]. Additionally, the lower part of a cone-shaped structure stabilizes the movement of particles by receiving the force from the bottom to the top, allowing effective mixing and movement near the center [36]. Therefore, it is confirmed from this study that the cone-shaped bottom design can optimize the flow phenomenon inside the FBR.

However, with the reduction in the inlet diameter and the increase in the angle of the cone-shaped bottom, the dead zone increased (Figure 8b,c). This indicates that the smaller diameter of the inlet increases the flow rate of the fluid, causing it pass through the reactor quickly, thereby failing to secure a sufficient reaction time and reducing the available area where particles can be effectively mixed [37]. Consequently, this leads to the problem of particles settling in certain areas, forming a dead zone. In addition, if the lower diameter of the fluidized bed reactor is small, mixing and dispersion at the inlet become difficult, hindering the uniform distribution of seed particles and reactants. Therefore, it is important to select an optimal diameter by considering the flow characteristics of the inlet. Furthermore, experimental confirmation of the FBR configuration with a lower angle of 12.6° and inlet diameter of 29 mm showed the best fluoride removal (86.6%), which is nearly 5% higher than the value obtained for the original configuration. 

**Figure 9:**
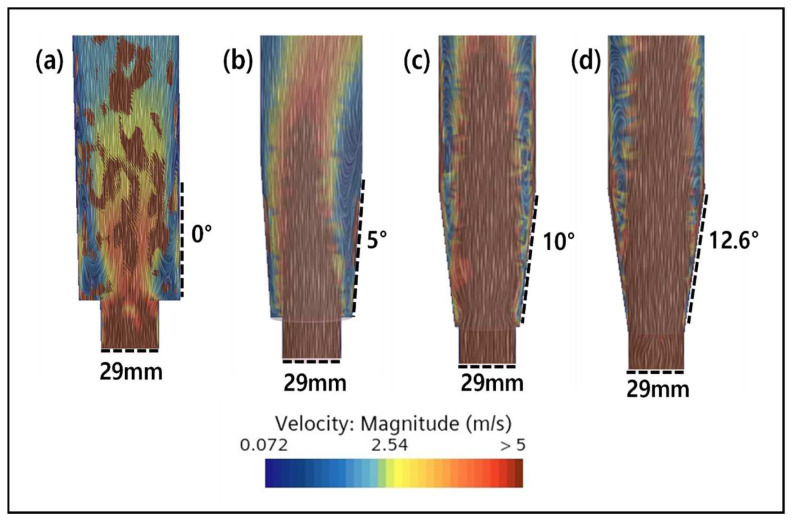
Effect of the bottom angle on mixing inside the FBR. Simulation results for (**a**) 0°, (**b**) 5°, (**c**) 10° and (**d**) 12.6° bottom angle.

The effect of changing the reactor bottom angle from 0° to 5°, 10° and 12.6° on reactor mixing is shown in Figure 9. The CFD simulation results clearly show that the mixing improved with the increase in the bottom angle as the dead zone gradually reduced. Particularly for the configurations with 10° and 12.6° angles, the dead zone in the reactor decreased significantly. In addition, there was almost no speed deviation for both these configurations, indicating that a uniform vertical rotation flow was being formed within the reactor. These results indicate that a uniform flow of particles in the reactor will be formed to prevent the precipitation of particles, and the optimal condition of 12.6°, which is the maximum angle at the bottom, was selected.

**Figure 10:**
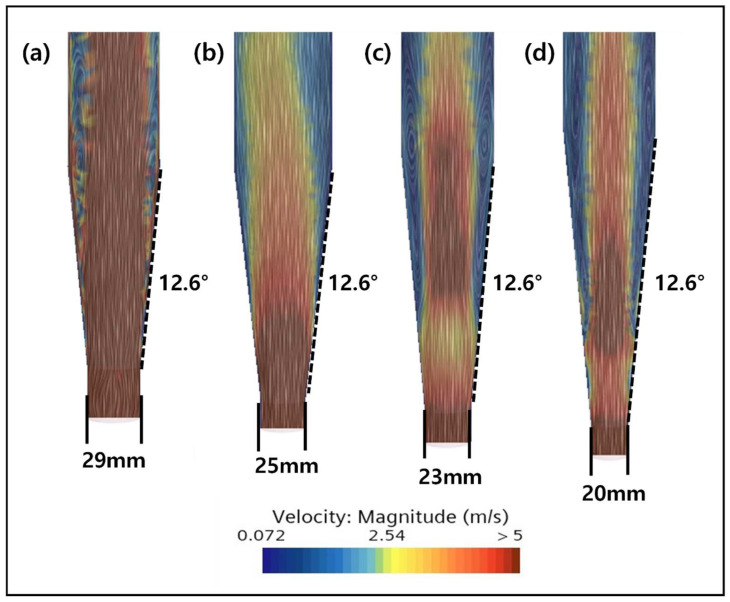
Effect of the inlet diameter on mixing inside the FBR. Simulation results for a (**a**) 29 mm, (**b**) 25 mm, (**c**) 23 mm and (**d**) 20 mm inlet diameter.

In the subsequent experiment, the inlet diameter of the reactor was reduced from 29 mm to 25 mm, 23 mm, and 20 mm by keeping the bottom angle fixed at 12.6°. From the results, it can be observed that the dead zone increases as the diameter of the inlet becomes smaller (Figure 10). This indicates the influence of the inlet diameter on the mixing inside the FBR. The principle of flow in the reactor is that the fluid flowing from the bottom rises to the top, and the rising flow moves in concentric circles in all directions and descends again along the wall [34]. Accordingly, the larger the inlet diameter, the relatively closer the distance between the bottom and the wall, so most of the relative inflow flow descends along the wall, resulting in smooth flow. This phenomenon is reflected in the CFD results, showing the best mixing for a wider inlet diameter. Therefore, an inlet diameter of 29 mm was chosen as optimal for smooth particle flow inside the FBR. 

**Figure 11:**
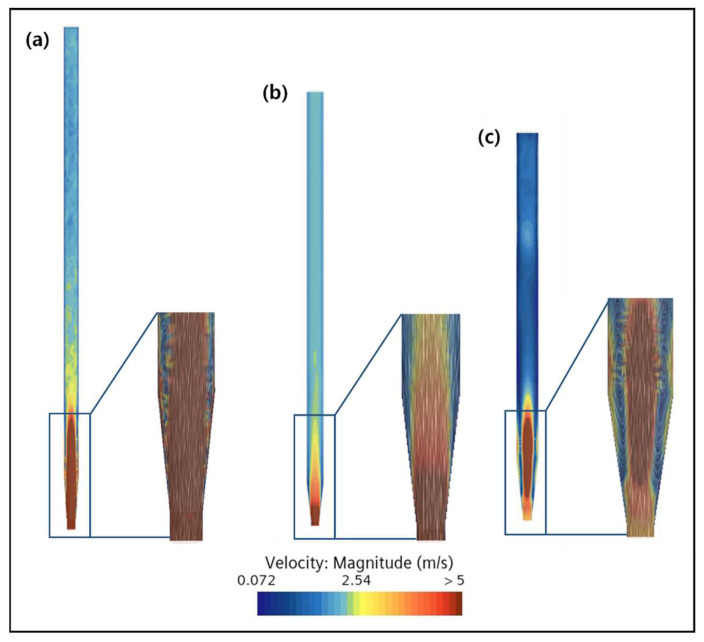
Effect of the width-to-height ratio of the reactor on mixing inside the FBR. Simulation results for a (**a**) 1:43, (**b**) 1:23 and (**c**) 1:15 width-to-height ratio.

The effect of the width-to-height ratio of the reactor on proper mixing was determined using CFD, and the result obtained is shown in Figure 11. The inlet diameter and bottom angle were kept fixed at 29 mm and 12.6°, respectively. The results showed that the best particle mixing was observed for the reactor with a 1:43 width-to-height ratio, which corresponds to the conventional reactor with a width of 50 mm and a height of 2150 mm used during the study. The dead zone gradually increased from this first configuration to the other two reactors, with width-to-height ratios of 1:23 and 1:15, respectively, indicating poor mixing inside these reactors. In the first reactor, a smooth flow of particles can be observed due to the narrow width of the reactor (Figure 11a). Additionally, it is believed that maintaining a constant fluid direction can enhance effective particle mixing by stabilizing the fluid flow at the bottom of the reactor and reducing the possibility of particle stagnation. The increase in width in the other two reactors caused instability in the flow pattern. This can be more clearly observed in the third configuration, with a width of 70 mm and a height of 1040 mm, where large-sized dead zones appeared inside the reactor due to a 20% increase in the width compared to the original reactor. Moreover, it was confirmed that the maximum particle height decreased due to the increase in the width despite having the same amount of seed particles as the original reactor. This indicates that, at the same flow rate, the pressure at the bottom of the reactor increases as the width of the reactor increases, requiring a larger flow rate to maintain the particles flowing at the same height. Therefore, in order to maintain the smooth mixing of particles inside the FBR, it is efficient to design the width of the reactor to be narrow. However, this drawback can be overcome by adjusting the flow rate as per the requirement based on the actual reactor conditions.

## 3. Materials and Methods

### 3.1. Materials Used

The chemicals used as sources for the fluoride and calcium sources in this work were hydrofluoric acid (55%, Hoimyung Waterzen, Suncheon, Republic of Korea) and calcium chloride (35%, Hoimyung Waterzen, Republic of Korea). The composition of the synthetic wastewater was made up using the following chemicals: glucose (≥98.5%, MB Cell, Republic of Korea), ammonium hydroxide (28.2%, Mallinckrodt, Hazelwood, MO, USA), sodium chloride (99%, Samchun, Pyeongtaek-si, Republic of Korea), silicon dioxide (100%, Daejung, Republic of Korea) and tungsten powder (99.9%, Daejung, Republic of Korea). For the pH adjustment, 1 M NaOH (95%, Samchun, Republic of Korea) and 1 M H_2_SO_4_ (95%, Samchun, Republic of Korea) were used.

### 3.2. Continuous Experimental Setup and Operation 

**Figure 12:**
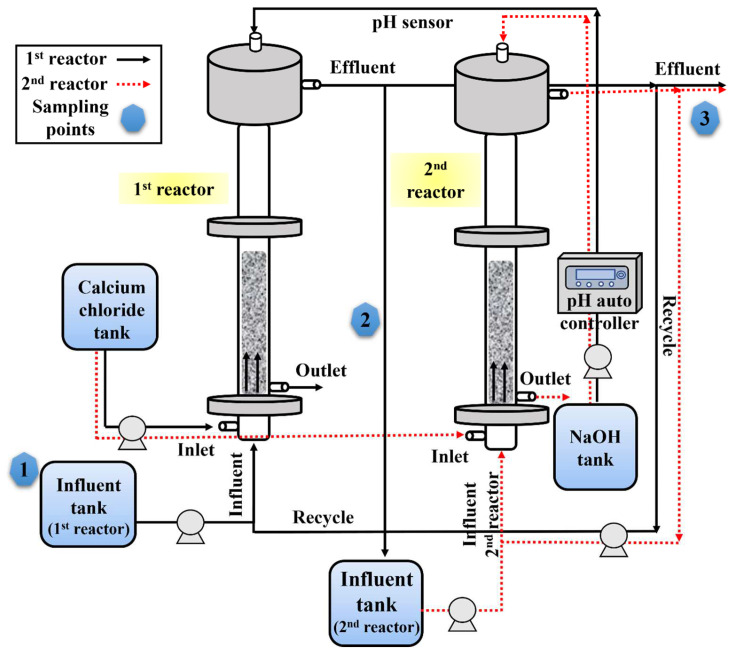
Experimental setup showing the single-stage and two-stage fluidized bed reactors used for continuous fluoride removal and recovery during this study.

In this study, a laboratory-scale fluidized bed reactor (FBR) of 3.44 L volume was manufactured using acrylic material and used for the fluoride treatment and calcium fluoride crystallization, as depicted in Figure 12. A detailed description of the reactor setup is provided in a previous work [14]. The second-stage reactor was similar in size and shape to the first reactor, including all the inlet and outlet ports (Figure 12). The second reactor was filled with silica seed materials of the required volume. The inflow and recirculation flow to the second reactor are distinctively identified by using red dotted lines in the figure. The sampling locations for collecting samples to and from the rector(s) are shown in the figure (Figure 12). 

During continuous experiment, fluoride-containing simulated wastewater was introduced to the reactor at an influent flow rate of 16.5 L/day and discharged accordingly. The synthetic wastewater used in this study was similar to semiconductor industry wastewater, according to Sim et al. [10], and had the following characteristics: pH 2.2, conductivity 5850 µs/cm, F^−^ 460 mg/L, NH^4+^-N 11.4 mg/L, SO_4_^2−^ 280 mg/L, total phosphorus (TP) 2.5 mg/L, SiO_2_ 10.3 mg/L, Al^3+^ 0.70 mg/L, Fe^2+^ 21.7 mg/L, and Mg^2+^ 0.1 mg/L. The HRT and upflow velocity were maintained at 5 h and 20 m/h, respectively. These selected operating parameters were based on the optimal operating conditions derived from our prior batch experiments [14]. A 10-fold diluted 35% CaCl_2_ reagent was continuously injected into the reactor at a rate of 0.29 mL/min to maintain the desired calcium-to-fluoride ion ratio in the reactor. The pH was maintained at the optimal value of 6 using a pH auto controller.

To monitor the fluoride removal efficiency, treated water samples were filtered once daily using a 0.45 μm GFC filter, with half of each sample being filtered. Subsequently, the fluoride ion concentration in both the unfiltered and filtered samples was measured to determine the fluoride removal rate and calcium fluoride crystallization efficiency. Furthermore, to track the crystal growth, the variation in seed height was monitored daily. The fluoride removal was calculated according to the following equation:

(1) $$Fluoride\;removal\;\left(\%\right)=\frac{{F^{-}}_{in}-{F^{-}}_{ef}}{{F^{-}}_{in}}\times 100$$

where F^−^_in_ and F^−^_ef_ are the influent and effluent fluoride concentration from the FBR, respectively.

### 3.3. Process Improvement Strategies

In order to determine if increasing the calcium concentration in the single-stage FBR process could improve the fluoride removal, the influent calcium concentration was increased to meet a [Ca^2+^]/[F^−^] ratio of 0.7, 0.75 and 0.8. Later, to overcome the hurdles associated with excessive calcium addition, a two-stage FBR process (Figure 12) was envisioned. In this two-stage FBR process, the effluent from the first reactor was used as the influent for the second stage. All the other conditions, including the reactor configurations, remained the same as described for the single-stage process. Different parameters key to the performance of the FBR for fluoride removal were optimized in this study. Firstly, as the influent fluoride concentration was low compared to the first reactor, the influent calcium concentration in the second reactor was optimized by varying the [Ca^2+^]/[F^−^] ratio to 0.4, 0.45, 0.5, 0.55, 0.6, 0.65, 0.7, 0.75 and 0.8. The [Ca^2+^]/[F^−^] ratio in the first stage was kept fixed at 0.55. In the next step, the HRT was optimized for the two-stage process by varying the HRT in the following manner (1st reactor + 2nd reactor): 5 h + 1 h, 4 h + 1 h, 3 h + 2 h, 3 h + 1 h, and 2 h + 2 h. 

### 3.4. CFD Guided Optimization of FBR Configuration

The CFD simulation was divided into four stages: geometry and flow domain, boundary and initial conditions, meshing (grid generation), and simulation. The boundary and initial conditions used in this study are shown in Table 2. The Lagrangian-multiphase equation can analyze the interaction between the solid region of the crystal and the liquid region of the wastewater, as well as the flow rate and pressure of each phase. Therefore, an equation that simultaneously analyzes two or more phases was included. 

In the case of the equations, the continuity equation and momentum equation were calculated using the k-epsilon model for turbulence generated due to a strong flow velocity. This model can be considered suitable for structural fluid analysis because it is an equation that provides a good compromise in terms of the accuracy and stability in general-purpose simulation. The resulting equation is as follows (Equation (2)).

Equation for kinetic energy (K):

(2) $$\frac{\alpha (\rho K)}{\alpha t}+\frac{\alpha (\rho u_{i}K)}{\alpha x_{i}}=\frac{\alpha }{\alpha x_{i}}\left[\left(µ+\frac{{µ}_{t}}{\sigma_{K}}\right)\frac{\alpha K}{\alpha x_{i}}\right]+P_{K}-\rho_{Є}$$

where ρ is the density of the fluid, t is the time, u_i_ is the velocity component, xi is the coordinate, μ is the dynamic viscosity, μ_t_ is the turbulent viscosity, σ_K_ is the modeling constant of the turbulent viscosity, and P_K_ is the kinetic energy. 

Equation for kinetic energy consumption rate (ε):

(3) $$\frac{\alpha (\rho_{Є})}{\alpha t}+\frac{\alpha (\rho u_{i}Є)}{\alpha x_{i}}=\frac{\alpha }{\alpha x_{i}}\left[\left(µ+\frac{{µ}_{t}}{\sigma_{Є}}\right)\frac{\alpha K}{\alpha x_{i}}\right]+C_{1Є}\frac{Є}{K}P_{K}-C_{2Є}\rho \frac{{Є}^{2}}{K}$$

where Є represents the turbulence energy (dissipation), σ_ε_ represents the modeling constant of the turbulence energy, and C_1ε_ and C_2ε_ represent the modeling constants. Afterwards, for the particle analysis, the analysis was performed using the Lagrangian method, as shown in the equation below, and the drag force (*F*_*D*_), lift force (*F*_*L*_), and gravity force were considered the forces acting on particles. The equation is as shown below (Equations (4)–(6)).

(4) $$\vec{F_{Di}}=C_{D}\frac{1}{2}\rho \left|u_{i}-u_{p,i}\right|(u_{i}-u_{p,i})A_{p}$$

(5) $$C_{D}=\alpha_{1}+\frac{\alpha_{2}}{R{Є}_{\rho }}+\frac{\alpha_{3}}{R{{Є}^{2}}_{\rho }}$$

(6) $${RЄ}_{\rho }=\frac{\rho \left|u_{i}-u_{p,i}\right|d_{\rho }}{µ}$$

where *dp* and *Ap* refer to the diameter and cross-sectional area of the particle. The particle diameter was 0.5 mm, the density was 2650 kg/m^3^, and it was assumed to be silica sand. *CD* is the drag coefficient, and *a*_1_, *a*_2_, and *a*_3_ are constants in the range of each constant *R*Є*p* number. *R*Є*p* refers to the Reynolds number of the particle.

(7) FLi→=mρ2Kvf1/2ρSijρpdp(SlkSkl)14(uj−up,j)

where *m*_*p*_ and *u*_*p*_ mean the mass and speed of the particle, and Li and Ahmadi’s equation, which is a generalization of the equation called Saffman’s lift, was used. The constant K is 2.594, and *ρ*_*p*_ and *ν*_*f*_ are the density of the particle and the fluid, respectively. This means the kinematic viscosity coefficient.

(8) $$\vec{F_{gi}}=m_{\rho }g_{i}$$

where gi means the force based on the law of universal gravitation.

The reactor configuration used in this study is depicted in Figure S1, along with all the dimensions. The FBR had a total height of 2150 mm and a width of 50 mm. The silica particles were filled up to 500 mm in height. The height of the lower part of the reactor was 95 mm and the fluid inlet part was 15 mm high and 29 mm in diameter. In order to verify the validity of the developed CFD program, a comparative study between the experimental and model-predicted results concerning the increase in the seed bed height at different upflow velocities was conducted. The different upflow velocities used for this experiment were as follows: 10 m/h, 20 m/h, 30 m/h, 40 m/h, 50 m/h, and 60 m/h. In this numerical analysis, particle analysis was conducted after excluding the energy loss due to the flow rate and the possibility of crystal shape deterioration. Once the validity of the developed CFD model was established, an experiment was conducted to understand the effect of the FBR configuration (bottom-part angle and inlet diameter) on mixing inside the reactor. For this purpose, three different configurations were chosen: first, the existing reactor with an inlet diameter of 29 mm; second, a new FBR with a lower angle of 12.6° and an inlet diameter of 29 mm; and third, another FBR with a lower angle of 19.1° and an inlet diameter of 20 mm (Figure S2). 

Further studies were conducted to identify the optimal design conditions using the optimized CFD-DEM model. Three design conditions were optimized: (i) the shape of the bottom of the reactor (according to the reactor bottom angle), (ii) the diameter of the reactor inlet, and (iii) the ratio of the width to height of the reactor. For the first experiment, the bottom angle of the reactor was increased from 0° to 5°, 10°, and 12.6° while keeping the inlet diameter fixed at 29 mm. Following this study, an experiment was conducted to study the effect of the inlet diameter by varying it from 29 mm to 25 mm, 23 mm and 20 mm. The width-to-height ratio was examined for three different ratios, namely, 1:43, 1:23, and 1:15. The reactor configurations for each of these experimental conditions are shown in Figure S3.

### 3.5. Characterization of the CaF_2_ Crystals

The calcium fluoride crystals formed inside the FBR were characterized using different instrumental techniques, namely, SEM-EDX, XRD, FTIR and ICP-OES. The SEM-EDX analysis was performed with an SEM instrument (Model Vega3, Tescan, Brno, Czech Republic) using a single calcium fluoride crystal retrieved from the FBR. The structural composition and crystallinity of the CaF_2_ crystals were measured using a high-resolution X-ray diffractometer (HR-XRD, Model D8 advance, Bruker, Billerica, MA, USA), and the scans were performed within the range of 05° to 90°. A Fourier transform infrared spectrometer (FT-IR, Model Spectrum 3, Perkin Elmer, Waltham, MA, USA) was used to determine the functional groups of the crystal structure. Compositional analysis of the CaF_2_ crystal was carried out with an inductively coupled plasma optical emission spectrometer (ICP-OES Model G8014AA, Agilent, Santa Clara, CA, USA). Analysis of silica seed as the control was similarly performed with all these instruments mentioned. 

### 3.6. Analytical Methods 

Analysis of the ionic components, including fluorine, calcium and sulfate, was performed using an ion chromatograph (IC, Model Ion chromatography-mass spectrometer, Metrohm, Herisau, Switzerland). All the samples were filtered using a 0.45 μm GFC filter prior to their analysis. The pH was measured using a pH meter (Model ST300, Ohaus, Parsippany, NJ, USA).

## 4. Conclusions

The fluoride bed crystallization process applied in this study demonstrated remarkably high fluoride removal over an extended period of time under a continuous mode of operation. The best results in terms of both the fluoride removal and crystallization efficiency were achieved within 40 days of reactor operation. However, prolonged reactor operation beyond 50 days led to a decline in performance. The CaF_2_ crystals recovered from the FBR showed high similarity with the standards, indicating their high purity. Further effort to improve the process efficiency through different approaches showed positive outcomes. Among the different methods explored, the two-stage FBR process showed promising results, achieving effluent fluoride concentrations in compliance with the regulatory standard of 15 mg/L. The CFD-based simulation technique was used to optimize the FBR configuration, revealing that further improvement in reactor performance can be achieved by modifying the reactor bottom shape, inlet diameter, and reactor width-to-height ratio. These optimized configurations, i.e., 12.6° bottom angle, 29 mm inlet diameter and 1:43 width-to-height ratio, showed the best mixing condition inside the FBR, which could result in better reactor performance. 

## Figures and Tables

**Table 1:** Elemental analysis of the silica seed and calcium fluoride crystals using ICP-OES.

Element	Silica Seed (%)	CaF_2_—70 Day (%)
Sulfate (S)	0.14	0.18
Aluminum (Al)	0.34	0.27
Calcium (Ca)	0.34	53.2
Fluoride (F)	1.53	44.4
Copper (Cu)	0.01	0.00
Iron (Fe)	0.70	0.56
Potassium (K)	0.27	0.36
Magnesium (Mg)	0.12	0.05
Sodium (Na)	0.38	0.47
Phosphate (P)	0.13	0.00
Silica (Si)	94.81	0.51
Zinc (Zn)	0.04	0.00

**Table 2:** Conditions used for the CFD flow analysis.

Condition (Unit)	Value
Liquid density (kg/m^3^)	997.561
Dynamic viscosity (Pa-s)	8.8871 × 10^−4^
Cp (cal/g.k)	0.998
Inlet velocity (m/s)	4.7 × 10^−4^
Temperature (°C)	25
Outlet pressure (Pa)	0
Solid density (kg/m^3^)	2650
Solid diameter (mm)	0.5
Number of grains	20,000
Particle count	5000

## Data Availability

Data will be made available on request.

## Supplementary Materials

The following supporting information can be downloaded at: https://www.mdpi.com/article/10.3390/ijms25094646/s1.
