# Supplementary material for: Process Intensification for Enhanced Fluoride Removal and Recovery as Calcium Fluoride Using a Fluidized Bed Reactor
Source: Int J Mol Sci. 2024 Apr 24;25(9):4646. doi: 10.3390/ijms25094646 (PMC11083762; doi:10.3390/ijms25094646)
Supplement: Supplementary file 1 [file ijms-25-04646-s001.zip › ijms-2957951-supplementary.pdf]

Supplementary material

Process intensification for enhanced fluoride removal and recovery as calcium fluoride using fluidized bed reactor

Arindam Sinharoy, Ga-Young Lee, Chong-Min Chung*

Department of Environmental Science & Biotechnology, Jeonju University, Jeonju 55069, Republic of Korea

* Corresponding author (C.M. Chung).

Tel.: +82632202286; E-mail address: cmchung@jj.ac.kr

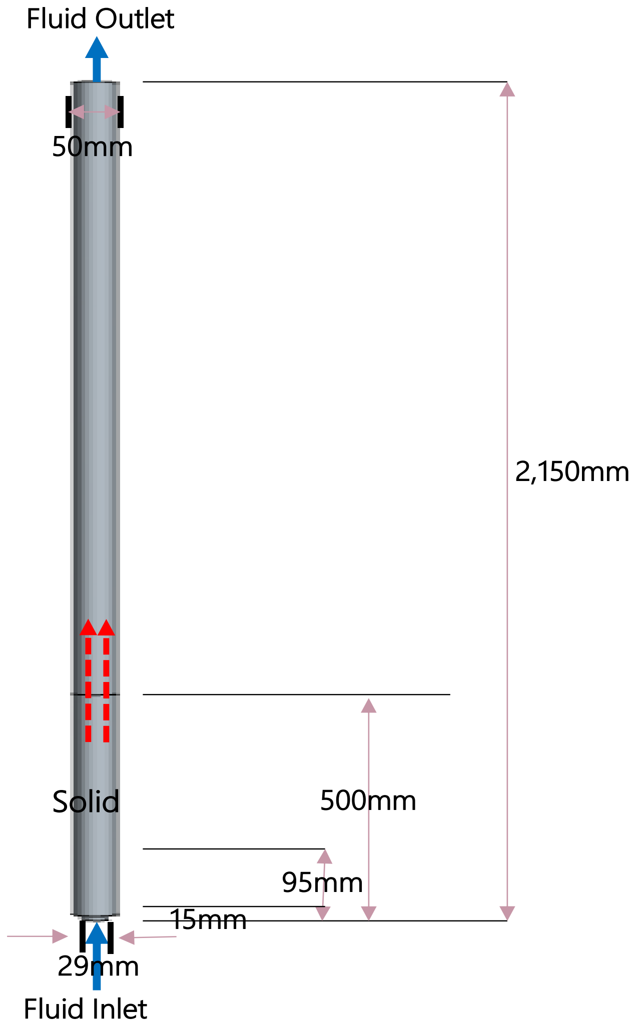

Figure S1. Configuration of reactor used for CFD numerical analysis.

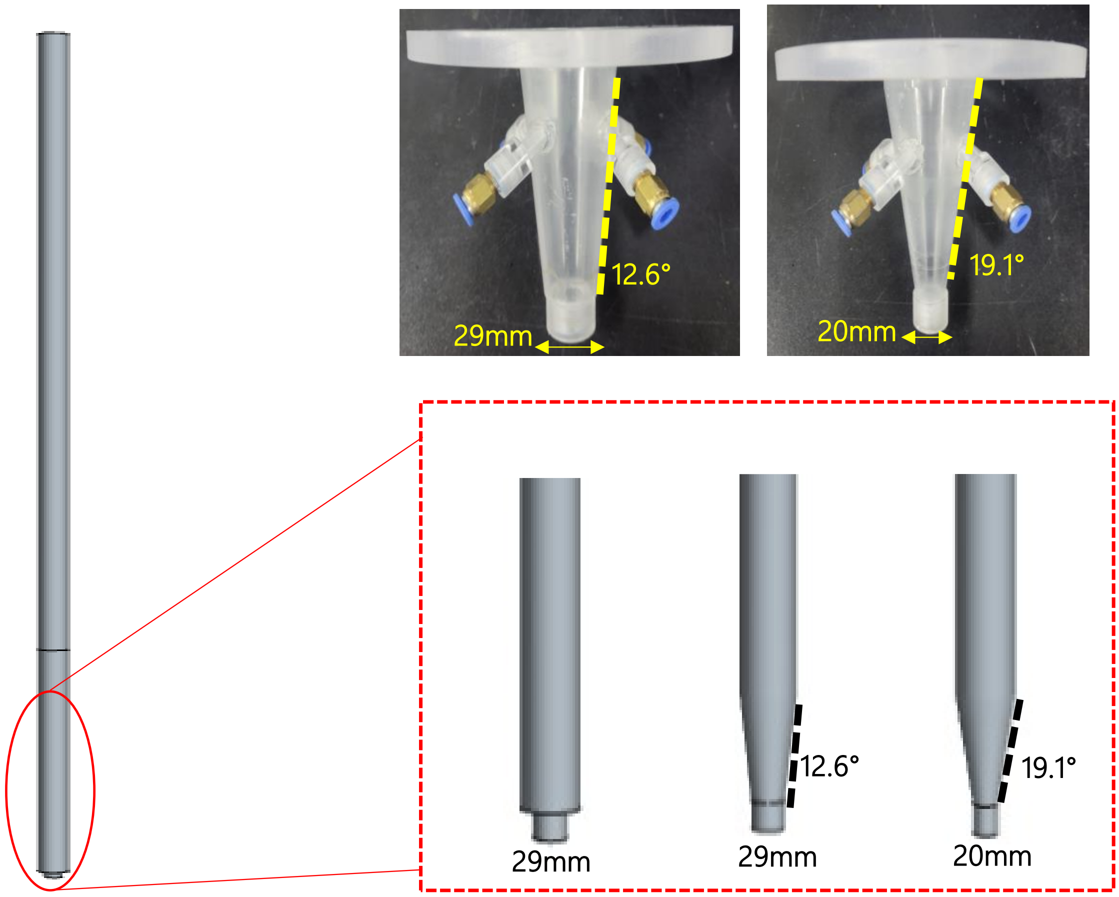

Figure S2. Configuration taken for CFD simulation under different conditions along with actual images of the reactor bottom.

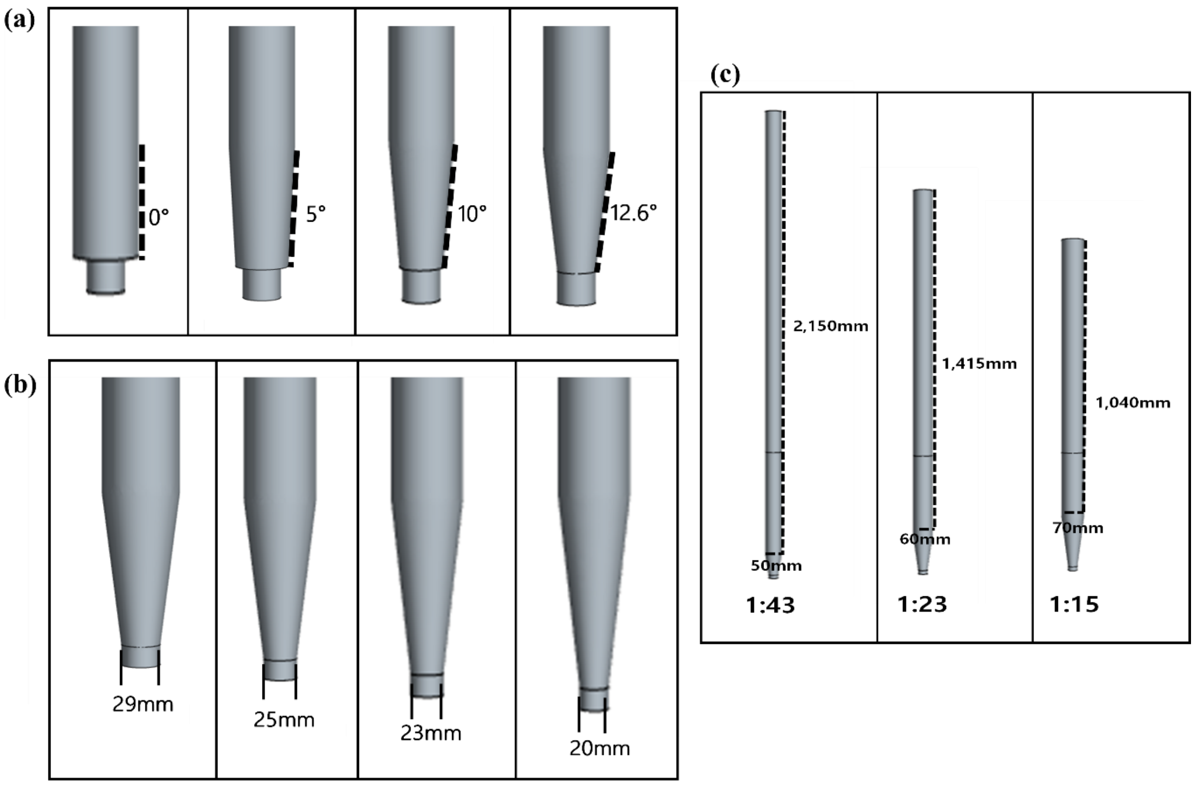

Figure S3. Different reactor configuration taken for CFD simulation to optimize shape of the FBR treating fluoride containing wastewater. (a) different shape of the bottom of the reactor (according to the reactor bottom angle), (b) different reactor inlet diameter, and (c) different width to height ratio of the reactor.

Calculation of CaF_2 purity

The purity of CaF_2 crystals = Total amount of the calcium fluoride crystals (assuming it to be 100 g) – amount of calcium present in the crystals with compounds other than CaF_2 (g) – all the other elements detected in ICP-OES apart from Ca and F (g)

The purity is calculated by first measuring the other compounds as given below:

Amount of Ca (in mol): 53.2 g/ (40 g/mol) = 1.33 mol

Amount of F (in mol): 44.4 g/ (19 g/mol) = 2.336 mol

As per the chemical formula for 2.336 mol of F 1.168 mol of Ca will be present in CaF_2.

Hence the remaining amount of Ca is: 1.33 mol – 1.168 mol = 0.162 mol x 40 g/mol = 6.48 g

The purity of CaF_2 crystals: 100 g – 6.48 g – (0.18 g + 0.27 g + 0.56 g + 0.36 g + 0.05 g + 0.47 g + 0.51 g) = 91.12 g which will be **91.12 % purity** as total amount of the calcium fluoride crystals is assumed to be 100 g.
